# The Contribution of Brainstem and Cerebellar Pathways to Auditory Recognition

**DOI:** 10.3389/fpsyg.2017.00265

**Published:** 2017-03-20

**Authors:** Neil M. McLachlan, Sarah J. Wilson

**Affiliations:** Melbourne School of Psychological Sciences, University of MelbourneMelbourne, VIC, Australia

**Keywords:** auditory, pathway, recognition, cognition, cerebellum

## Abstract

The cerebellum has been known to play an important role in motor functions for many years. More recently its role has been expanded to include a range of cognitive and sensory-motor processes, and substantial neuroimaging and clinical evidence now points to cerebellar involvement in most auditory processing tasks. In particular, an increase in the size of the cerebellum over recent human evolution has been attributed in part to the development of speech. Despite this, the auditory cognition literature has largely overlooked afferent auditory connections to the cerebellum that have been implicated in acoustically conditioned reflexes in animals, and could subserve speech and other auditory processing in humans. This review expands our understanding of auditory processing by incorporating cerebellar pathways into the anatomy and functions of the human auditory system. We reason that plasticity in the cerebellar pathways underpins implicit learning of spectrotemporal information necessary for sound and speech recognition. Once learnt, this information automatically recognizes incoming auditory signals and predicts likely subsequent information based on previous experience. Since sound recognition processes involving the brainstem and cerebellum initiate early in auditory processing, learnt information stored in cerebellar memory templates could then support a range of auditory processing functions such as streaming, habituation, the integration of auditory feature information such as pitch, and the recognition of vocal communications.

## Introduction

Despite extensive evidence for cerebellar involvement in auditory conditioned behaviors in animals (Aitkin and Boyd, 1975, 1978; Perrett et al., 1993; Lingenhöhl and Friauf, 1994; Medina et al., 2000; Ohyama et al., 2003; Thompson and Steinmetz, 2009), this paper describes the first neurocognitive model to integrate cortico-ponto-cerebellar pathways into the structure and functions of human auditory pathways. In particular, the neurophysiology of the auditory ponto-cerebellar pathways is first described in relation to conditioned behaviors in animals. Models of these networks are then integrated with the Object-Attribute Model (OAM) of auditory processing (McLachlan and Wilson, 2010), and supporting evidence from neuroimaging and lesion studies for cerebellar involvement in a wide range of human auditory behaviors is provided.

The importance of the cerebellum for motor control has been known since the early 19th century (Ghez and Fahn, 1985; Houk et al., 1996; Glickstein and Doron, 2008). Over a century later Marr proposed that, “the purpose of the cerebellum is to learn motor skills, so that when they have been learned a simple or incomplete message from the cerebrum will suffice to provoke their execution” (Marr, 1969). About the same time Ito (1970) showed that the output from the cerebellar cortex was inhibitory, suggesting that the cerebellum regulated the execution of motor sequences rather than initiated them. Detailed physiological data on cerebellar neural architecture (Eccles, 1967) has since been used by researchers to propose a wide range of information processing models to explain motor control and conditioned and unconditioned reflexes (Marr, 1969; Fujita, 1982; Thompson, 1986; Kawato et al., 1987; Moore et al., 1989; Houk et al., 1993; Wolpert et al., 1998; Medina and Mauk, 2000; Ito, 2006). The common feature of these models is that the cerebellar cortex learns temporal sequences of sensory inputs associated with particular events that enable the precise timing of behaviorally relevant motor responses.

The cerebellum is connected to the rest of the brain by three peduncles; (i) the inferior peduncle receives input from the inferior olivary nucleus and the spinocerebellar and vestibular systems, (ii) the middle peduncle receives input from the pons, and (iii) the superior peduncle largely projects to the thalamus (Glickstein and Doron, 2008; Granziera et al., 2009). The principal cerebellar input from the motor system is via the red nucleus to the inferior olivary nucleus and then the inferior peduncle (Kawato et al., 1987; Wolpert et al., 1998; Granziera et al., 2009). In contrast, inputs to the middle peduncle from the pons originate from prefrontal and parietal cerebral pathways (Ramnani, 2006; Glickstein and Doron, 2008; Ito, 2008; Strick et al., 2009), although afferent sensory inputs have also been documented (Graybiel, 1974; Aitkin and Boyd, 1975; Glickstein and Doron, 2008). In particular afferent auditory inputs from the inferior colliculus (IC) and cochlear nucleus (CN) to the pons (Snider and Stowell, 1944; Aitkin and Boyd, 1975, 1978) and the inferior olivatory nucleus (Huang et al., 1982) have been reported. The principal cerebellar outputs from the superior peduncle are to the red nucleus which relays information to premotor nuclei in the brainstem, and to the thalamus which then connects broadly to the cerebral cortex and to the amygdala (Wolpert et al., 1998; Ramnani, 2006; Ito, 2008; Strick et al., 2009).

## Behavioral Adaptation in Primitive Auditory Networks

The cerebellum is one of the oldest and most structurally conserved brain regions in vertebrates (Weaver, 2005; Apps and Hawkes, 2009), and in many mammalian species, it contains more than three times as many neurons as the neocortex (Herculano-Houzel, 2010). Iguanas, one of the most evolutionarily ancient lizard species, possess frequency selective hearing (Manley, 2002) and their auditory pathways include the brainstem, thalamus and cerebellum. Like all reptiles they lack neocortex (Wyneken, 2007) and many of the higher auditory processing centers found in primates. While marine iguanas do not communicate vocally, they are capable of distinguishing the predator alarm calls of mockingbirds from other mockingbird songs to initiate escape and alert behaviors (Vitousek et al., 2007). This behavior points to the early evolution of sound recognition abilities in primitive terrestrial vertebrates, and the involvement of sub-cortical auditory processing pathways in sound recognition.

Habituation to predictable sounds is another evolutionarily ancient behavioral response to auditory signals. Neural networks in the dorsal CN of mice (and even in the electrosensory systems of fish) have been shown to adapt to supress responses to predictable stimuli (Roberts and Portfors, 2008). Lateral inhibitory fields adapt to sharpen spectral features in neural representations (or templates) of commonly occurring signals and amplify their edges. This makes these networks particularly sensitive to stimulus driven activation that differs from learnt templates for commonly occurring signals, so they can supress responses to these stimuli without loss of sensitivity to novel stimuli (Tzounopoulos et al., 2004; Roberts and Portfors, 2008). Roberts and Portfors (2008) made the observation that the neural architecture of these dorsal CN circuits is very similar to the architecture of cerebellar circuits. However, the CN does not include a network capable of storing temporal sequences and operates on spectral information only.

Startle responses to acoustic stimuli are common in terrestrial vertebrates. In rats, startle responses have very short latencies of about 5 ms and habituation is frequency specific (Fleshler, 1965; Lingenhöhl and Friauf, 1994), indicating the presence of a rapid neural pathway with the capacity to learn spectral information. To account for the speed of the acoustic startle response Lingenhöhl and Friauf (1994) described a neural circuit comprising only three synapses in which the CN innervates giant neurons in the pons, which in turn, innervates cranial and spinal motor neurons. Consistent with this, Aitkin and Boyd (1978) described a subset of cells (15%) in the dorsolateral pontine nucleus with onset latencies to auditory stimuli as short as 3 ms that likely receive direct input from the CN (Huang et al., 1982). Adaptation of dorsal CN response fields enables habituation to common sounds (Tzounopoulos et al., 2004; Roberts and Portfors, 2008) and so could account for the frequency selectivity of habituation of startle responses to acoustic stimuli, while the pons connectivity to motor circuits provides specificity of the motor response to stimuli that elicit startle responses.

In rabbits, auditory conditioning of eye-blinks can be achieved by repeated paired presentations of an initially neutral stimulus, such as a tone, with puffs of air (Perrett et al., 1993; Medina et al., 2000; Ohyama et al., 2003; Thompson and Steinmetz, 2009). Rabbits can be taught to blink with high accuracy at any time between 100 and 600 ms after the onset of a tone at a specific frequency, with the accuracy and number of blink responses diminishing at longer time intervals up to about 3 s (Ohyama et al., 2003). In contrast, the minimum onset latency of an eye-blink in response to a puff of air in rabbits is about 25–40 ms (Thompson and Steinmetz, 2009). Lesions of the cerebellar cortex permanently abolish the adaptive timing of blinks, leaving only frequency specific reflex responses to tones with short and relatively fixed delays (Perrett et al., 1993; Ohyama et al., 2003). In humans, auditory conditioning of the eye-blink reflex with a delay of 720 ms between the conditioned and unconditioned stimuli has been observed in healthy people, but not in patients with damage to cerebellar structures (Daum et al., 1993). This indicates that the cerebellar cortex plays a role in learning accurate temporal relationships between acoustic and tactile stimuli in humans and other mammals.

In summary, very primitive auditory neural circuits in the CN are able to habituate behavioral responses to commonly occurring acoustic signals. In contrast, auditory conditioned eye-blinks in rabbits involve the timed initiation of a behavior associated with a specific auditory spectrum. Correct timing of conditioned eye-blink responses requires the involvement of the cerebellar cortex. Finally, the ability of marine iguanas to recognize mockingbird alarm calls likely involves a recognition pathway that includes the cerebellar cortex, since mockingbird song typically comprises large frequency sweeps over short temporal pulses that require both spectral and temporal information to categorize (Logan and Fulk, 1984).

### Auditory Information Processing Architecture of the Cerebellum

**FIGURE 1:**
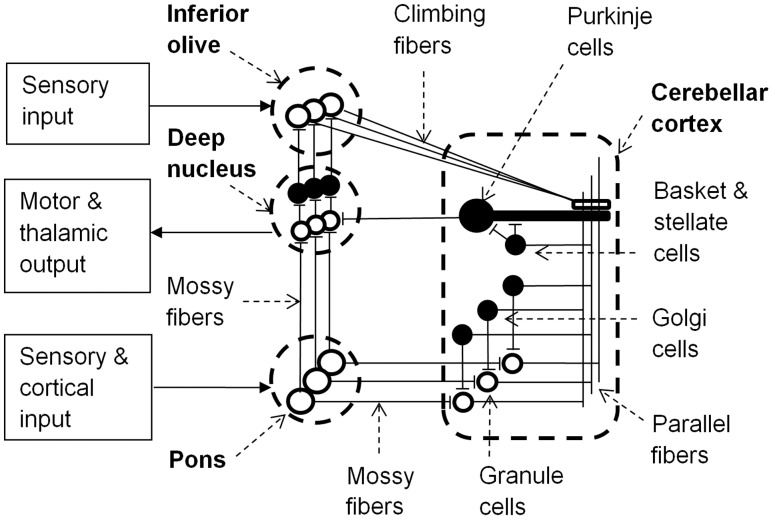
**Learning circuit of the cerebellum (black neurons are inhibitory)**. Input to the pons becomes associated with motor and thalamic outputs via the deep nucleus and is also relayed via granule cells and parallel fibers to Purkinje cells. Granule cells have varying latencies allowing specific timing of inhibition of the deep nucleus by Purkinje cells. Golgi, basket and stellate cells refine spatio- (or spectro-) temporal response fields of Purkinje cells. Sensory input to the inferior olive can alter Purkinje cell synaptic strengths to parallel fiber inputs and the inferior olive is inhibited by activation of the deep nucleus. Adapted from Yeo and Hesslow (1998) and Sacchetti et al. (2005).

The cerebellar cortex includes granule, basket, stellate, and Golgi cells. These cells are connected by the axons of granule cells that form long parallel fibers (about 3 mm in length) that excite profusely branched dendrites of Purkinje cells (**Figure 1**). Inhibitory basket and stellate cell axons run on either side of the excitatory parallel fibers that act on Purkinje cells, and so fine tune neural response fields in the network. Purkinje cells inhibit neurons in the deep cerebellar nuclei, thereby regulating the excitatory drive that these neurons receive from the pons. Purkinje cells project to the closest deep cerebellar nuclei, so the lateral hemispheres project to the dentate nucleus, the intermediate cortex project to the globose and emboliform nuclei, and the vermis projects to the fastigial nucleus (Granziera et al., 2009). Small regions of the cerebellar cortex with similar somatotopic receptive fields form microcomplexes, which project to one region of the deep cerebellar nuclei (Apps and Garwicz, 2005). These microcomplexes are repeated throughout the cerebellum generating a vast network of feed-forward information processing units interconnected by parallel fibers in the cerebellar cortex. Furthermore microcomplexes located in different regions of cerebellar cortex may be enervated from the same inferior olive neuron and project to the same group of motor neurons, allowing parallel processing of sensory inputs to achieve integrated motor responses (Apps and Garwicz, 2005).

In each microcomplex, neurons in the deep cerebellar nuclei and the cerebellar cortex receive inputs from mossy fibers that originate in the pons. The pons receives inputs from the brainstem and cerebral cortex, and so mossy fiber inputs to granule cells convey both direct sensory information and cerebral contextual information. Purkinje cells also receive input from excitatory climbing fibers that originate in the inferior olive. The inferior olive receives excitatory input directly from the brainstem and indirectly from the parietal cortex via the red nucleus (Oka et al., 1979). Conjunctive stimulation of climbing and mossy fibers at different firing rates can either excite or inhibit Purkinje cells, thereby providing learning feedback mechanisms for the network by changing the weights of parallel fiber synapses to Purkinje cells (Ito et al., 1982; Medina et al., 2000). This alters the probability of a Purkinje cell firing to a particular set of cortical, sensory and inter-cerebellar inputs, and so with repeated exposure the cerebellar pathways could learn appropriate behavioral responses to sensory and/or proprioceptive information in relation to error messages from the inferior olive and the specific environmental/behavioral context provided by the cortical input via the pons (Ito, 2008).

Medina and Mauk (2000) and Medina et al. (2000) developed a computer simulation of auditory conditioning of the eye-blink reflex in rabbits to investigate how temporal information is processed in the cerebellar cortex. They used physiologically realistic populations and connectivity of cerebellar cell types that were modeled with relatively simple “leaky integrator” model neurons. Auditory input to the pons was based on recordings in cats of mossy fiber responses to pure tones by Aitkin and Boyd (1975) in which about 4% of fibers displayed either onset or tonic responses to a particular tone. Consistent with the proposition that the pons associates spectral patterns of neural activation with motor reflexes, and transmits these spectral response patterns to the cerebellar cortex (Medina et al., 2000), Purkinje cell responses in the cerebellar vermis of cats display a wide variety of frequency response patterns (Aitkin and Boyd, 1975). Furthermore, these response patterns are stable over wide ranges of stimulus intensity, as would be required for recognition mechanisms that are independent of stimulus loudness. Axon and terminal degeneration in the dorsolateral pontine nucleus after lesion of the IC indicated that the IC was a source of the afferent auditory input to the mossy fibers (Aitkin and Boyd, 1978).

In the model by Medina et al. (2000) the cerebellar cortex consisted of a layer of granule cells connected to Purkinje cells and a second layer of inhibitive Golgi cells. The modeled cerebellar cortex spontaneously evolved temporal specificity when the synaptic weights between granule and Purkinje cells decreased in strength if active in the presence of a climbing fiber input, and increased in strength if active in the absence of a climbing fiber input (Medina et al., 2000). After training over several 100 trials various populations of the model granule cells developed temporal response characteristics that involved specific periods of enhanced or depressed firing rates (Medina et al., 2000). As a result, different sets of granule cells would be active at different times during the presentation of the conditioned stimulus (tone), and the inhibitory action of Purkinje cells on the deep nuclei could be timed to allow a blink reflex at the expected time of the unconditioned stimulus (e.g., an air puff; Medina et al., 2000). This model contrasts significantly with earlier models of cerebellar timing computation that propose a plurality of neural delay lines associated with specific motor or sensory functions (Eccles, 1967; Braitenberg et al., 1997). The principle difficulties with delay line models of cerebellar computations include: (1) the very short time domain over which sequences could be encoded (less than 200 ms), (2) the very precise neural timing required of Purkinje cells to synchronize with the velocity of neural conduction, and (3) the excitatory capacity of parallel fibers to activate Purkinje cells as required in delay line models (Braitenberg et al., 1997). In contrast to neural delay line models, the model proposed by Medina et al. (2000) provides the possibility that temporally imprecise sequences of pons activations associated with cortical inputs (such as phoneme or word sequences) with relatively long delay times may be learnt by the cerebellum.

**FIGURE 2:**
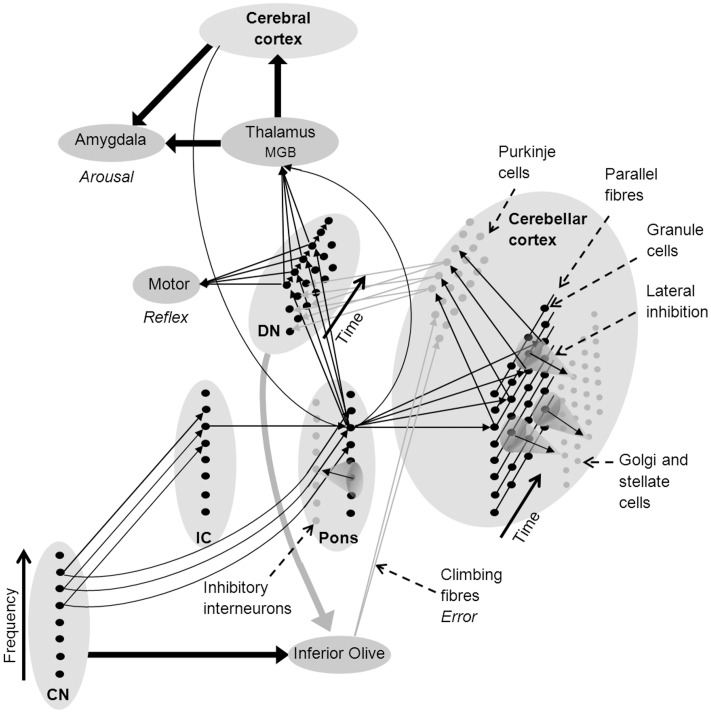
**Network architecture in the cerebellum to support sound recognition and associated auditory processes (see text for a detailed explanation)**. Fine arrows denote neural connections involved in sound recognition and thick arrows denote connectivity between brain nuclei and regions (black denotes excitatory connections and gray inhibitory connections). CN, cochlear nucleus; DMGB, dorsal medial geniculate body; DN, deep nuclei of the cerebellum; and IC, inferior colliculus.

Like motor reflexes, learnt fear responses to acoustic stimuli are rapid and frequency specific. They have been shown to evoke shifts in the frequency responses of neurons in the medial geniculate body (MGB) of the thalamus (Weinberger, 2011). The thalamus projects to the amygdala, which is associated with autonomic arousal in response to aversive stimuli under moderation from the anterior cingulate and other frontal cortical areas (Critchley, 2005). The thalamus is enervated by the deep nuclei of the cerebellum and the pons (Paré et al., 1990; Reese et al., 1995), forming a neural circuit that can rapidly learn to recognize auditory stimuli that are paired with pain or threat, generate autonomic arousal, and adapt these learnt associations (**Figure 2**) (Critchley, 2005; Sacchetti et al., 2005; Weinberger, 2011).

Many researchers have suggested that the consistency of neural architecture throughout the cerebellum indicates that it undertakes similar types of information processing on all its inputs, regardless of whether they are sensory, proprioceptive, or cortical in origin (Courchesne and Allen, 1997; Ramnani, 2006; Thach, 2007; Ito, 2008). As a consequence, existing neurocognitive models of cerebellar function should be consistent with existing models of auditory processing. **Figure 2** shows a network model of the cerebellar cortex that adapts the cerebellar neural architecture proposed by Medina et al. (2000) according to the proposition that sound recognition occurs in sub-cortical pathways suggested in the OAM of auditory processing by McLachlan and Wilson (2010). In **Figure 2**, spectral patterns of afferent auditory information are initially recognized in the CN-pons neural circuit. Multiple arrows between the CN, pons and deep nucleus in **Figure 2** represent auditory input to giant cells in the pons from the CN that drive startle reflexes with very short latencies and low spectral specificity (Fleshler, 1965; Lingenhöhl and Friauf, 1994). Auditory inputs to the cerebellar cortex from the IC (via the pons) have a broad range of spectral and temporal response patterns including a small proportion of finely tuned tonic mossy fiber responses (Aitkin and Boyd, 1978) that provide higher spectral specificity than CN inputs over time (represented in **Figure 2** as a single arrow from the IC).

Pons neurons can excite the deep nucleus to initiate motor reflexes, and the thalamus to initiate both autonomic arousal via the amygdala, and the association of symbolic or multi-modal identities in the cerebral cortex. Purkinje cells in the cerebellar cortex also receive excitatory innervation from the pons via mossy and parallel fibers, and from the inferior olive via climbing fibers, and then project to the deep nucleus where they inhibit feed-forward motor and other responses if they receive an unexpected sensory input (Konnerth et al., 1990; Medina et al., 2000). The deep nuclei also send projections to the inferior olive which may inhibit error messages when a firing sequence is progressing according to expected sensory inputs (Best and Regehr, 2009).

The association of spectrotemporal templates with identities in the secondary auditory cortex likely constitutes the second stage of sound identification (McLachlan and Wilson, 2010; McLachlan, 2011). This is consistent with previous research that identified contributions of both auditory feature processing and categorical knowledge formation in sound recognition mechanisms (Ballas, 1993). It is also consistent with neurophysiological data on sound recognition mechanisms (the “what” pathway) recorded in the anterolateral belt region of the auditory cortex of rhesus monkeys (Rauschecker and Tian, 2000). Rauschecker and Tian (2000) found neurons in this region that responded much more strongly to full bandwidth reproductions of monkey calls than spectrally filtered reproductions. These neurons also responded strongly to the complete temporal sequence of a monkey call, but only weakly to the first part of the call, and not at all to the second part of the call. In other words, neurons in the anterolateral belt of the auditory cortex preferentially respond to the specific temporal order of spectral information associated with a particular monkey call. Similar neurophysiological data has also been observed in the prefrontal cortex of monkeys (Averbeck and Romanski, 2006), suggesting that the semantic meaning of calls with particular spectrotemporal properties is stored in anterolateral belt regions of the auditory cortex and processed in frontal cortical regions.

## The Cerebellum in Human Auditory Processing

The cerebellum contains two somatotopic representations (homunculi) in primates (Snider and Eldred, 1951; Grodd et al., 2001; Imamizu et al., 2003; Manni and Petrosini, 2004), and in Macaque monkeys, magnetic resonance tractography has shown that the dominant cerebral input to the cerebellum is from the motor areas (Ramnani et al., 2006). However, in humans, the lateral regions of the neocerebellum are by far the largest part of the cerebellum and receive input from the prefrontal and parietal areas of the cerebral cortex via the pons (Kelly and Strick, 2003; Ramnani et al., 2006; Strick et al., 2009). Stoodley et al. (2012) reported functional magnetic resonance imaging (fMRI) data for humans that showed that finger-tapping activated right cerebellar lobules IV, V and VIII along with the sensorimotor cerebral cortices. In contrast, verb generation, mental rotation, and working memory tasks activated the lateral neocerebellar lobules VI (Crus I) and VII (Crus II), along with prefrontal and parietal cerebral cortices. These data are consistent with the idea that implicit learning in the cerebellum may involve cognitive processes in the lateral Crus I and II lobules that are more abstract than basic integration of sensory and motor information (Imamizu et al., 2003; Konoike et al., 2012). This idea is further supported by recent evidence of fMRI activation in these lateral cognitive regions for purely visual sensory processing tasks such as the recognition of emotions in facial images (Baumann and Mattingley, 2012), and the motion of visual bars on a monitor (Kellermann et al., 2012). These visual functions may be supported by afferent visual connections to the pons via the superior colliculus, lateral geniculate body and pretectal area in the mid brain of mammals that were described in the 1970s (Graybiel, 1974).

Specific regions in the lateral cerebellum in humans, particularly in the left Crus I area, are consistently activated in brain imaging studies involving auditory tasks ranging from passive listening to pure tones or clicks, to various types of active auditory discrimination of intensity, duration, location, pitch, timbre and speech (Petacchi et al., 2005; Callan et al., 2007; Sens and de Almeida, 2007; Wilson et al., 2009). Tasks involving temporal processing of auditory stimuli have shown that individuals with lateral cerebellar lesions could not accurately perceive the difference between ‘longer’ and ‘shorter’ acoustic tone bursts (Ivry et al., 1988), and cerebellar involvement in this task was later confirmed in normal individuals in a fMRI study by Mathiak et al. (2004). Similar studies have also indicated that the lateral cerebellum is involved in classification of speech stimuli based on temporal dynamics (Ackermann et al., 2004). Finally Konoike et al. (2012) found fMRI activation in left Crus I of the cerebellum, the superior temporal gyrus, the inferior parietal lobes and the inferior frontal gyrus during a memory task requiring the encoding of a rhythmic phrase. Performance of the rhythm after a short time delay resulted in bilateral activation of the lobule VI regions of the cerebellum and in the cerebral motor areas. However, no activation was observed in the cerebellum during the maintenance of the rhythm in working memory. These data are consistent with involvement of the cortico-ponto-cerebellar system (including the frontal and parietal cortices) for encoding rhythmic information, but only the frontal regions of the cerebral cortex for maintaining a memory trace of the rhythm in working memory.

### Speech and Sound Recognition

The rapid enlargement of the ventrolateral portion of the cerebellum in conjunction with the inferior frontal region of the cortex in humans suggests selective evolution of prefrontal input to the human cortico-ponto-cerebellar system (Ramnani, 2006), driven largely by increasing demands of language processing (Leiner et al., 1989). Murdoch (2010) suggested that the cortico-ponto-cerebellar system likely underpins the phonological loop, a short term memory store for phonological information that is supported by articulatory rehearsal of information (Baddeley et al., 1998), while Kotz and Schwartze (2010) postulated that the cerebellum plays an important role in the temporal regulation of cortical phonemic processing in relation to motor templates for speech production via the basal ganglia and thalamus. More recently, Schwartze and Kotz (2016) proposed that precise temporal processing in the cerebellum regulates cortical integration of auditory information through oscillatory feedback loops between cerebellar and cerebral cortices. They suggest that an event-based temporal representation of the speech signal could predict auditory dynamics and so regulate and optimize cortical attentional resources.

Lesion, neurophysiological and imaging studies have highlighted the role of the right lateral cerebellum in a range of non-motor aspects of language (Schlösser et al., 1998; Marien et al., 2001; Xiang et al., 2003; Frings et al., 2006; Callan et al., 2007; Stoodley et al., 2012; Moberget et al., 2014). For example, agrammatic speech can follow focal lesions that are relatively circumscribed to the right lateral cerebellum (Zettin et al., 1997), and repetitive transcranial magnetic stimulation over the right lateral cerebellum disrupted language function (Lesage et al., 2012). Given the homogeneity of cerebellar neural architecture (Apps and Garwicz, 2005), Moberget and Ivry (2016) explored whether predictive forward models that have been developed to explain cerebellar motor control would also be consistent with the demands of speech processing. This implies that non-spatial ‘what’ information can be processed by cerebellar neural architecture, and so they provide an account of how non-spatial error signals provided by climbing fibers in the cerebellar cortex (**Figure 1**) might enable learning of semantic information.

In response to reports of auditory neural plasticity in the thalamus of rats (Weinberger, 2011), McLachlan and Wilson (2010); McLachlan (2011), and McLachlan N. et al. (2013) speculated that the spectrotemporal template matching mechanisms proposed in the OAM may reside in the MGB. However, the vast neural resources available in the pons and lateral cerebellum, and their direct connectivity to the afferent pathways of the auditory brainstem and thalamus make them ideally suited to this role. Given spectral integration of auditory inputs may occur in the pons prior to cerebellar processing (Snider and Stowell, 1944; Aitkin and Boyd, 1975, 1978), the ponto-cerebellar pathways are ideally suited to undertake matching of auditory inputs with spectrotemporal memory templates, and associate these templates with symbolic identities in secondary auditory cortex (Davis and Johnsrude, 2003; Schwartze and Kotz, 2016).

**FIGURE 3:**
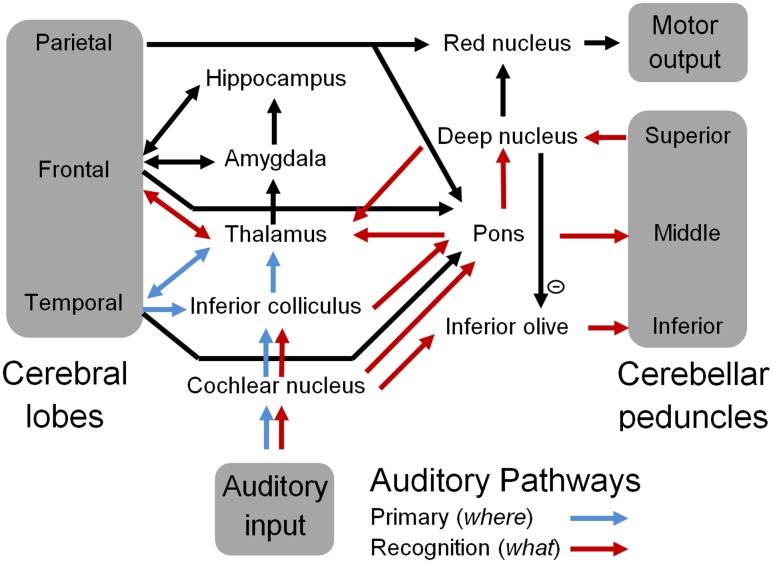
**A schematic representation of the auditory pathways that include the cerebellum**. Blue arrows represent well-known primary auditory pathways, and red arrows represent the proposed sound recognition pathways that comprise sub-cortical aspects of the “what” auditory pathway including the cerebellum.

**Figure 3** is a schematic representation of the ‘what and where’ auditory pathways (Arnott et al., 2004) that were initially discussed in the OAM (McLachlan and Wilson, 2010). These have now been expanded to include the cerebellum as the neural substrate for spectrotemporal template matching of speech and other auditory information. According to the updated OAM shown in **Figure 3**, the medial MGB of the thalamus relays information from the pons and deep nuclei of the cerebellum to the secondary auditory cortex (specifically the anterolateral belt region) for sound identification in the auditory ‘what’ pathway (Rauschecker and Tian, 2000; Arnott et al., 2004; McLachlan and Wilson, 2010). The dorsal MGB projects in parallel to the caudolateral belt region of the auditory cortex, which in turn projects to the parietal cortex as part of the auditory ‘where’ pathway (Rauschecker and Tian, 2000; McLachlan and Wilson, 2010). Various authors have proposed a multidimensional memory representation of acoustic stimuli including pitch, location, and loudness (Grau and Kemler-Nelson, 1988; Gomes et al., 1995; McLachlan and Wilson, 2010) associated with the ‘where’ pathway (Arnott et al., 2004), and bound by an overall gestalt such as an identifiable timbre that has been encoded in the ‘what’ pathway (McLachlan and Wilson, 2010).

By proposing that spectral integration of auditory information for sound recognition occurs in the pons rather than the cerebral cortex, the extended OAM reduces the role of the cerebellum in sound and speech processing to the learning and recognition of temporal sequences of pons inputs. Similarly, integration of visual information in the pons (via the superior colliculus) would allow the cerebellum to process temporal sequences of visual shapes. This allows the cerebellum to readily integrate synchronous multimodal sensory inputs, since all sensory modalities are represented by sequences of pons neural activations. Cortico-ponto-cerebellar connectivity then allows specific spectrotemporal and/or multimodal templates to be associated with symbolic identities that are encoded in the cerebral cortex. In turn this will allow commonly occurring sequences of these higher level identities such as words and phrases to be learnt from cerebral inputs to the pons, and enable subsequent automatic processing in the cerebellum (Ramnani, 2006; Ito, 2008; Argyropoulos, 2016), as suggested by neuroimaging studies (Moberget et al., 2014) and neurophysiological studies (Argyropoulos, 2011; Lesage et al., 2012).

The proximity of speech recognition to speech articulation mechanisms in the cerebellum would allow articulatory motor control maps to integrate closely with spectrotemporal phoneme recognition templates (Kotz and Schwartze, 2010), thereby enhancing speech learning and perception, particularly when only partial information for either task is available (Lindblom, 1996). Furthermore, cerebellar disorders may give rise to ataxic dysarthria, which is characterized by distinct articulatory and phonatory deficits (Ackermann et al., 2007). The location of speech recognition templates in the lateral cerebellum may therefore contribute to the large size increase of the lateral cerebellum as humans evolved speech capacity (Ramnani et al., 2006). A wide range of behavioral deficits have been observed in patients with cerebellar lesions including language deficits, and impairment of executive functions, abstract reasoning and spatial cognition (Schmahmann and Sherman, 1998; O’Halloran et al., 2012). Consistent with findings that associate cerebellar damage with neuropsychological disorders, communication deficits in low functioning autism spectrum disorder (ASD) have been associated with decreased density of Purkinje cells in the cerebellar cortex, while high functioning ASD has been associated with enlarged cerebellar volumes and increased gray matter (Salmond et al., 2007; Amaral et al., 2008).

The pons and deep nuclei of the cerebellum project to the MGB, which regulates inputs to the auditory cortex from the IC. Inhibition and/or potentiation of MGB neurons according to spectrotemporal patterns stored in cerebellar sound recognition templates may cause rapid plasticity of response fields of primary auditory cortex neurons that are enervated by the ventral MGB (Paré et al., 1990; Fritz et al., 2005; Weinberger, 2011). Rapid plasticity of the response fields of primary auditory cortex neurons could enable the streaming and integration of auditory features that are associated with an expected or attended acoustic source (David et al., 2012; Zion Golumbic et al., 2013). Fritz et al. (2010) recorded response patterns from neurons in the frontal cortex of ferrets that rapidly adapted during trained behaviors to encode task-relevant information through enhanced responses to behaviorally conditioned tones and suppressed responses to other tones. Consistent with the proposition that frontal cortex modulates primary auditory cortex neural sensitivity, adaptation of primary auditory cortex neural responses during and after behavior was similar to the adaptation of the frontal cortex neurons that were recorded simultaneously. Thus the cerebellar network could play an important role in regulating the auditory ‘where’ pathway through its connectivity with frontal regions of the cerebral cortex as shown in **Figure 3**. Finally, rarely described efferent pathways from the cerebellar nuclei to the pons (Rand, 1954; Achenbach and Goodman, 1968) may provide a fast network for priming expected pons neurons, and so streaming auditory information based on predictions from cerebellar processing.

### Learning

Association of a recognized sound with a symbolic identity in the cerebral cortex could occur via the neural pathway from the deep nucleus to the dorsal MGB and the auditory association areas (**Figure 3**). Ito (2008) suggested that prefrontal and parietal cortical connections with the inferior olive via the red nucleus could support cerebellar learning of purely cognitive tasks, and in support of this proposition, electrical stimulation of the parietal cortex in cats has been shown to activate climbing fibers from the inferior olive (Oka et al., 1979). Error messages from the inferior olive are used to alter Purkinje cell synaptic connections to parallel fibers within the cerebellar cortex. In motor control tasks these error messages may include pain, somatosensory and visual information (Wolpert et al., 1998; Ramnani, 2006; Ito, 2008). In sound recognition these messages may also be produced by complete inhibition of the deep nucleus by Purkinje cells when recognition mechanisms fail. The inferior olive receives inhibitory innervation from the deep nucleus, so that it is not active during successful recognition processing, and is most active when an unexpected change occurs in the stimulus timing (Liu et al., 2008; Best and Regehr, 2009). The inferior olive also receives excitatory input from the CN, so if template matching fails in the cerebellum during stimulus presentation, the inferior olive could send error messages to the cerebellar cortex to adjust Purkinje cell synaptic strengths according to the stimulus properties. Inferior olive neurons are strongly electrically coupled by gap junctions (Llinas et al., 1974) and so tend to fire synchronously (Welsh et al., 1995). This may provide widespread coordination across cerebellar microcomplexes of precise temporal sequences, such as occurs during eye-blink conditioning (Van Der Giessen et al., 2008).

These learning mechanism may be sufficient to support learning to identify sounds without requiring error feedback from the prefrontal cortex, for which there is little neuroanatomical evidence to date. In other words, spectrotemporal templates in the ponto-cerebellar network might be organized by sensory and proprioceptive feedback, and simply labeled by association with symbolic and multimodal identities in the prefrontal cortex without cerebral cortical feedback to the inferior olive. Reptiles can learn complex auditory signals (Vitousek et al., 2007) without cerebral error feedback to the inferior olive, which could not evolve until after neocortex appeared in mammals. Feedback from cerebral cortex may also be too slow and largely redundant, since the cerebellar pathways can make rapid multisensory associations during motor planning (Wolpert et al., 1998).

The cortico-pontine-cerebellar pathway enables priming of cerebellar templates by cortical working memory and attentional mechanisms as described in Ramnani (2006) for motor control, and in the expanded OAM for sound recognition proposed in this review. According to **Figure 2**, activation of the pons by enervation from the association cortex in the absence of an auditory stimulus will activate a temporal sequence of neural activations in the deep nucleus. Activation of the deep nucleus could drive activation of the MGB in the thalamus in spectrotemporal sequences that would be similar to actually perceiving a well-remembered timbre, and lead to the experience of auditory imagination. This is consistent with the observation of patterns of cerebral fMRI activation associated with imagining sounds that are similar to those observed when hearing sound, albeit without activation of primary auditory cortex (Zatorre and Halpern, 2005). Moreover, fMRI activation of the left lateral regions of the cerebellum, and frontal and parietal cerebral areas was greater when people imagined well-known melodies compared to perceiving well-known melodies (Herholz et al., 2012), and non-motor related cerebellar activation was associated with covert singing when contrasted with overt singing (Kleber et al., 2007). This is consistent with the association of left lateral cerebellar activation with pitch discrimination rather than motor control (Holcomb et al., 1998), and suggests that pitch and timbral templates are stored in this region of the cerebellum.

The echoic trace was described in the OAM (McLachlan and Wilson, 2010) as a buffer of stimulus driven activation in the thalamus that enables review of recent auditory information when sound identification is ambiguous, or an individual makes a conscious decision to attend to detailed auditory information. However, in the extended OAM outlined in this review, the echoic trace is proposed to be the sequence of pons neural activations. Amygdala activation associated with increased arousal due to hypervigilance or the failure of recognition mechanisms activates the hippocampus to store sensory information (Phelps, 2004). Storage of the sequence of pons activations, rather than the full sensory fields that caused these activations, will substantially reduce the amount of information that must be stored in the hippocampus. This will also allow that information to be readily integrated with other sensory information (Schwartz and Their, 1999), recalled through the primary auditory pathways by sequential activation of the pons neurons, or consolidated with long-term memory via connectivity to the ponto-cerebellar pathways (Datta et al., 2005).

### Music

McLachlan (2009, 2011) proposed that pitch was first approximated by both the frequency and the pattern of auditory nerve excitation, as in a harmonic template matching model of pitch (Terhardt et al., 1982), before being further refined by temporal processing of the stimulus waveform. According to the expanded OAM presented in **Figures 2** and **3**, initial spectral pitch processing would occur in the CN-IC-pons network. This is consistent with research that shows that the ponto-cerebellar network learns highly specific responses to frequency information from the auditory brainstem (Perrett et al., 1993; Medina et al., 2000; Ohyama et al., 2003), and the matching of learnt spectral templates to music stimuli in humans (McLachlan N. et al., 2013; McLachlan N.M. et al., 2013).

Pitch resolution is refined by waveform driven mechanisms in the IC to produce sharper frequency resolution after multiple stimulus periods (Langner and Schreiner, 1988; de Cheveigné, 2005; Meddis and O’Mard, 2006; McLachlan, 2009; McLachlan and Grayden, 2014). According to McLachlan (2009, 2011) the primed spectral pitch is associated with pitch dimension in a place code by neurons adjacent to the primary auditory cortex (Bendor and Wang, 2005). Lateral inhibition mechanisms in this network then operate as an adaptive filter, only allowing periodicity information from the IC that is consistent with the primed pitch to contribute to the pitch representation in short term memory. Consequently, as the frequency resolution of neural response patterns in the IC sharpen over multiple stimulus periods the pitch representation in auditory short term memory also sharpens. Furthermore, since the cerebellar cortex also receives input from the CN-IC-pons network over multiple stimulus periods, more refined frequency representations that develop over time in the IC may contribute to improved stimulus recognition over longer latencies. Efferent pathways from the deep nucleus of the cerebellum to the pons (Rand, 1954; Achenbach and Goodman, 1968) may contribute to refining pons firing patterns as pitch estimates become more precise over time.

Consistent with cerebellar involvement in pitch processing, Parsons et al. (2009) found that pitch discrimination thresholds of high functioning patients afflicted with varying degrees of global cerebellar degeneration performed on average, over five times poorer than controls, with performance proportional to the degree of cerebellar ataxia. Furthermore, a positron emission tomography (PET) study by Holcomb et al. (1998) found increased blood flow in the middle and left lateral cerebellum associated with the motor and decision components of a pitch recognition task, respectively. Hutchinson et al. (2003) subsequently reported that male musicians had larger cerebellar volumes relative to their total brain volume compared to non-musicians. This potentially represents structural adaptation to extended periods of music practice for enhanced motor control, and in light of Holcomb et al. (1998), for better pitch discrimination. Consistent with this, Abdul-Kareem et al. (2011) reported increased white matter volumes in the middle and superior cerebellar peduncles of musicians and Sokolov et al. (2014) reported diffusion tensor imaging evidence for a structural loop between the left cerebellum and the right superior temporal sulcus. Finally, pitch processing by absolute pitch musicians is faster than other musicians (Hsieh and Saberi, 2007), suggesting that they are able to use rapid recognition mechanisms in the CN-IC-pons network to identify standard pitches for which they have previously established fine pitch associations in auditory short term memory (Wilson et al., 2012).

Observed differences in pitch processing between musicians and non-musicians (Seither-Preisler et al., 2007; Kraus and Chandrasekaran, 2010) and musicians from different cultures (McLachlan N.M. et al., 2013) are consistent with the generation of long-term memory templates for musical stimuli in the ponto-cerebellar network through music training. Recent behavioral data shows that dissonance is experienced when musicians are presented with uncommon chords for which recognition mechanisms are likely to fail (McLachlan N. et al., 2013). Failure of cerebellar recognition mechanisms may lead to increased activation of the amygdala via activation of the thalamus by the deep nucleus (**Figures 2** and **3**). The amygdala also connects to the hippocampus and plays an important role in regulating autonomic arousal and the encoding of episodic memory (Poldrack et al., 2001) with additional input from the prefrontal cortex (Critchley, 2005). Increased hippocampal and parahippocampal activation has been associated with the experience of dissonance (Wieser and Mazzola, 1986; Blood et al., 1999), which is consistent with the activation of this pathway by failure of cerebellar recognition mechanisms for unfamiliar or incongruent musical stimuli (McLachlan N. et al., 2013).

In contrast, Blood et al. (1999) and Salimpoor et al. (2012) reported activation of the dopaminergic reward network when listeners reported feeling pleasure while listening to their favorite music. Activation of the dopaminergic brainstem pathways in humans has been shown for problem solving without explicit external rewards in other cognitive (non-musical) domains (Tricomi et al., 2006). The reward network was only activated by familiar music (Blood et al., 1999; Salimpoor et al., 2012), suggesting that successful predictions about the trajectory of musical features generated by cerebellar processing of familiar musical sequences may be involved as observed by Konoike et al. (2012) for rhythms. In rats, the nucleus accumbens receives input from frontal cortical regions that are enervated by the thalamus, and in turn, by the ponto-cerebellar pathways. The nucleus accumbens can either activate positively valanced reward, or negatively valanced dread behaviors, depending on the recognized stimulus and contextual information (Berridge and Kringelbach, 2013). When people listen to music, complex relationships between musical predictions based on well-known musical sequences may combine with episodic memories for peak personal experiences in frontal cortical regions to activate the reward network via the nucleus accumbens as observed by Blood et al. (1999) and Salimpoor et al. (2012).

### Embodied Cognition

The cerebellum is involved in purely cognitive processes such as mental and perceptual timing, learning and reproducing new word and rhythmic phrases, and the mental rehearsal of speech and other movements (Fiez et al., 1992; Gebhart et al., 2002; Ravizza et al., 2006; Konoike et al., 2012). Ramnani (2006) suggested that the difference between cerebral and cerebellar information processing may be that in cerebellar circuits both the context and the rule are integrated in the same internal representation, and can only be efficiently applied in that context, whereas cerebral circuits abstract rules and relationships and maintain them in working memory. This allows cerebral circuits to rapidly adapt to novel stimuli and circumstances, whereas the cerebellum gradually learns implicit associations over multiple presentations of stimuli.

Broca’s area in the inferior frontal cortical region in humans is important for learning sequences which contain abstract structures rather than fixed sequences (Dominey et al., 2003; Vandervert, 2011). It has a well-established role in the grammatical processing of phonological sequences (Roll et al., 2012), and has also been implicated in the processing of musical syntax (Maess et al., 2001). This suggests that commonly occurring sensory information may be learnt in patterns of neural connectivity in the cerebellum (templates) that are associated with conceptual identities stored in secondary auditory cortex via the thalamus (**Figure 3**), and arranged and manipulated in abstract grammars in Broca’s area. In this way, implicit sensori-motor memories stored in the cerebellum may enable the automation of well-rehearsed cognitive processes as a form of embodied cognition (Mahon and Caramazza, 2008). This could enable more efficient performance of cognitively demanding tasks by releasing cerebral neural resources from undertaking well-rehearsed processes so that they can monitor and adapt behavior based on broader behavioral and environmental perspectives (Ito, 2008). In other words, the cerebellum can undertake automatic processing of well-rehearsed sensory, cognitive and motor functions.

## Conclusion

Behaviors that are specific to a particular spectrotemporal feature of a sound may include its association with other sensory information or verbal labels, heightened autonomic arousal, startle and conditioned motor reflexes, and even spectral and temporal changes in the sensitivity of the auditory system itself. All of these behaviors are examples of neural plasticity associated with sound recognition, and have been observed in brain regions as early as the dorsal CN, where simple adaptation of auditory sensitivity for common sounds can occur (Tzounopoulos et al., 2004; Roberts and Portfors, 2008). Here we propose that more complex behaviors that include specific temporal properties of the stimulus involve the cerebellar cortex (Perrett et al., 1993; Ohyama et al., 2003), while cognitive tasks such as sound identification involve most of the auditory pathways including the anterior temporal lobe of the cerebral cortex (McLachlan and Wilson, 2010). The proposition that sound recognition commences in the cerebellar pathways is consistent with the initial premise of the OAM that sound recognition commences early in the auditory processing pathways (McLachlan and Wilson, 2010). In the expanded OAM, recognition of spectral features is proposed to occur in the CN-IC-pons network prior to recognition of temporal sequences of these features in the cerebellar cortex.

Overall, these observations point to the existence of an evolutionarily ancient sound recognition mechanism in the brainstem and hindbrain of terrestrial animals that is capable of implicit learning of spectrotemporal sequences of sound. The pons and deep nucleus of the cerebellum project to the thalamus that regulates the response fields of neurons in the primary auditory pathway. This could enable auditory information to be integrated and streamed according to the spectrotemporal properties of recognized sound sources. In turn, expectations about auditory information that are generated in the cerebral cortex may prime sound recognition mechanisms via the cortico-pontine-cerebellar pathway, leading to enhanced auditory sensitivity to behaviorally relevant sound sources. It should be noted, however, that cerebellar involvement in sound recognition may not be required when spectral information alone is sufficient to recognize sounds, or when accurate temporal processing is not essential.

## Author Contributions

NM was the principle author and initially developed the models described in the paper. SW refined and expanded the models and undertook detailed reviewing and editing of the text in collaboration with NM.

## Conflict of Interest Statement

The authors declare that the research was conducted in the absence of any commercial or financial relationships that could be construed as a potential conflict of interest.

## References

[B1] Abdul-Kareem I. A. Stancak A. Parkes L. M. Al-Ameen M. Alghamdi J. Aldhafeeri F. M. (2011). Plasticity of the superior and middle cerebellar peduncles in musicians revealed by quantitative analysis of volume and number of streamlines based on diffusion tensor tractography. *Cerebellum* 10 611–623. 10.1007/s12311-011-0274-1 21503593

[B2] Achenbach K. E. Goodman D. C. (1968). Cerebellar projections to Pons. Medulla and spinal chord in the albino rat. *Brain Behav. Evol.* 1 43–57. 10.1159/000125492

[B3] Ackermann H. Mathiak K. Ivry R. B. (2004). Temporal organization of “internal speech” as a basis for cerebellar modulation of cognitive functions. *Behav. Cogn. Neurosci. Rev.* 3 14–22. 10.1177/1534582304263251 15191639

[B4] Ackermann H. Mathiak K. Riecker A. (2007). The contribution of the cerebellum to speech production and speech perception: clinical and functional imaging data. *Cerebellum* 6 202–213. 10.1080/14734220701266742 17786816

[B5] Aitkin L. M. Boyd J. (1975). Responses of single units in cerebellar vermis of the cat to monaural and binaural stimuli. *J. Neurophysiol.* 38 418–429. 1127449 10.1152/jn.1975.38.2.418

[B6] Aitkin L. M. Boyd J. (1978). Acoustic input to the lateral pontine nuclei. *Hear. Res.* 1 67–77. 10.1016/0378-5955(78)90010-2 757232

[B7] Amaral D. G. Schumann C. M. Nordahl C. W. (2008). Neuroanatomy of autism. *Trends Neurosci.* 31 137–145. 10.1016/j.tins.2007.12.005 18258309

[B8] Apps R. Garwicz M. (2005). Anatomical and physiological foundations of cerebellar information processing. *Nat. Rev. Neurosci.* 6 297–311. 10.1038/nrn1646 15803161

[B9] Apps R. Hawkes R. (2009). Cerebellar cortical organization: a one-map hypothesis. *Nat. Rev. Neurosci.* 10 670–681. 10.1038/nrn2698 19693030

[B10] Argyropoulos G. P. (2011). Cerebellar theta-burst stimulation selectively enhances lexical associative priming. *Cerebellum* 10 540–550. 10.1007/s12311-011-0269-y 21451999

[B11] Argyropoulos G. P. (2016). The cerebellum, internal models and prediction in ‘non-motor’ aspects of language: a critical review. *Brain Lang.* 161 4–17. 10.1016/j.bandl.2015.08.003 26320734

[B12] Arnott S. R. Binns M. A. Grady C. L. Alain C. (2004). Assessing the auditory dual-pathway model in humans. *Neuroimage* 22 401–408. 10.1016/j.neuroimage.2004.01.014 15110033

[B13] Averbeck B. B. Romanski L. M. (2006). Probabilistic encoding of vocalizations in macaque ventral prefrontal cortex. *J. Neurosci.* 26 11023–11033. 10.1523/JNEUROSCI.3466-06.2006 17065444 PMC6674661

[B14] Baddeley A. Gathercole S. Papagno C. (1998). The phonological loop as a language learning device. *Psychol. Rev.* 105 158–173. 10.1037/0033-295X.105.1.158 9450375

[B15] Ballas J. A. (1993). Common factors in the identification of an assortment of brief everyday sounds. *J. Exp. Psychol.* 19 250–267. 10.1037/0096-1523.19.2.250 8473838

[B16] Baumann O. Mattingley J. B. (2012). Functional topography of primary emotion processing in the human cerebellum. *Neuroimage* 61 805–811. 10.1016/j.neuroimage.2012.03.044 22465459

[B17] Bendor D. Wang X. (2005). The neuronal representation of pitch in primate auditory cortex. *Nature* 436 1161–1165. 10.1038/nature03867 16121182 PMC1780171

[B18] Berridge K. C. Kringelbach M. L. (2013). Neuroscience of affect: brain mechanisms of pleasure and displeasure. *Curr. Opin. Neurobiol.* 23 294–303. 10.1016/j.conb.2013.01.017 23375169 PMC3644539

[B19] Best A. R. Regehr W. G. (2009). Inhibitory regulation of electrically coupled neurons in the inferior olive is mediated by asynchronous release of GABA. *Neuron* 62 555–565. 10.1016/j.neuron.2009.04.018 19477156 PMC3261724

[B20] Blood A. J. Zatorre R. J. Bermudez P. Evans A. C. (1999). Emotional responses to pleasant and unpleasant music correlate with activity in paralimbic brain regions. *Nat. Neurosci.* 2 382–387. 10.1038/7299 10204547

[B21] Braitenberg V. Heck D. Sultan F. (1997). The detection and generation of sequences as a key to cerebellar function: experiments and theory. *Behav. Brain Sci.* 20 229–277. 10.1017/S0140525X9700143X 10096998

[B22] Callan D. E. Kawato M. Parsons L. Turner R. (2007). Speech and song: the role of the cerebellum. *Cerebellum* 6 321–327. 10.1080/14734220601187733 17853077

[B24] Courchesne E. Allen G. (1997). Prediction and preparation, fundamental functions of the cerebellum. *Learn. Mem.* 4 1–35. 10.1101/lm.4.1.1 10456051

[B25] Critchley H. D. (2005). Neural mechanisms of autonomic, affective, and cognitive integration. *J. Comp. Neurol.* 493 154–166. 10.1002/cne.20749 16254997

[B26] Datta S. Saha S. Prutzman S. L. Mullins O. J. Mavanji V. (2005). Pontine-wave generator activation-dependent memory processing of avoidance learning involves the dorsal hippocampus in the rat. *J. Neurosci. Res.* 80 727–737. 10.1002/jnr.20501 15880522 PMC1224707

[B27] Daum I. Schugens M. M. Ackermann H. Lutzenberger W. Dichgans J. Birbaumer N. (1993). Classical conditioning after cerebellar lesions in humans. *Behav. Neurosci.* 107 748–756. 10.1037/0735-7044.107.5.748 8280385

[B28] David S. V. Fritz J. B. Shamma S. A. (2012). Task reward structure shapes rapid receptive field plasticity in auditory cortex. *Proc. Natl. Acad. Sci. U.S.A.* 109 2144–2149. 10.1073/pnas.1117717109 22308415 PMC3277538

[B29] Davis M. H. Johnsrude I. S. (2003). Hierarchical processing in spoken language comprehension. *J. Neurosci.* 23 3423–3431. 12716950 10.1523/JNEUROSCI.23-08-03423.2003 PMC6742313

[B30] de Cheveigné A. (2005). “Pitch perception models,” in *Pitch – Neural Coding and Perception*, eds Plack C. Oxenham A. Fay R. R. Popper A. N. (New York, NY: Springer), 169–233.

[B31] Dominey P. F. Hoen M. Blanc J. M. Lelekov-Boissard T. (2003). Neurological basis of language and sequential cognition: evidence from simulation, aphasia, and ERP studies. *Brain Lang.* 86 207–225. 10.1016/S0093-934X(02)00529-1 12921765

[B32] Eccles J. C. (1967). *The Cerebellum as a Neuronal Machine.* New York, NY: Springer 10.1007/978-3-662-13147-3

[B33] Fiez J. A. Petersen S. E. Cheney M. K. Raichle M. E. (1992). Impaired non-motor learning and error detection associated with cerebellar damage. *Brain* 115 155–178. 10.1093/brain/115.1.155 1559151

[B34] Fleshler M. (1965). Adequate acoustic stimulus for startle reaction in the rat. *J. Comp. Physiol. Psychol.* 60 200–207. 10.1037/h0022318 5832345

[B35] Frings M. Dimitrova A. Schorn C. F. Elles H. G. Hein-Kropp C. Gizewski E. R. (2006). Cerebellar involvement in verb generation: an fMRI study. *Neurosci. Lett.* 409 19–23. 10.1016/j.neulet.2006.08.058 17046160

[B36] Fritz J. Elhilali M. Shamma S. (2005). Active listening: task-dependent plasticity of spectrotemporal receptive fields in primary auditory cortex. *Hear. Res.* 206 159–176. 10.1016/j.heares.2005.01.015 16081006

[B37] Fritz J. B. David S. V. Radtke-Schuller S. Yin P. Shamma S. A. (2010). Adaptive, behaviorally gated, persistent encoding of task-relevant auditory information in ferret frontal cortex. *Nat. Neurosci.* 13 1011–1019. 10.1038/nn.2598 20622871 PMC2921886

[B38] Fujita M. (1982). Adaptive filter model of the cerebellum. *Biol. Cybern.* 45 195–206. 10.1007/BF00336192 7171642

[B39] Gebhart A. G. Petersen S. E. Thach W. T. (2002). “The role of the cerebellum in language,” in *Recent Developments in Cerebellar Research*, eds Highstein S. M. Thach W. T. (New York, NY: New York Academy of Sciences), 318–333. 10.1111/j.1749-6632.2002.tb07577.x 12582063

[B40] Ghez C. Fahn S. (1985). “The cerebellum,” in *Principles of Neural Science*, 2nd Edn, eds Kandel E. R. Schwartz J. H. (Amsterdam: Elsevier), 502–522.

[B41] Glickstein M. Doron K. (2008). Cerebellum: connections and functions. *Cerebellum* 7 589–594. 10.1007/s12311-008-0074-4 19002543

[B42] Gomes H. Ritter W. Vaughan H. G. Jr. (1995). The nature of preattentive storage in the auditory system. *J. Cogn. Neurosci.* 7 81–94. 10.1162/jocn.1995.7.1.81 23961755

[B43] Granziera C. Schmahmann J. D. Hadjikhani N. Meyer H. Meuli R. Wedeen V. (2009). Diffusion spectrum imaging shows the structural basis of functional cerebellar circuits in the human cerebellum in vivo. *PLoS ONE* 4:e5101 10.1371/journal.pone.0005101 PMC2659746 19340289

[B44] Grau J. W. Kemler-Nelson D. G. (1988). The distinction between integral and separable dimensions: evidence for integrality of pitch and loudness. *J. Exp. Psychol.* 117 347–370. 10.1037/0096-3445.117.4.347 2974862

[B45] Graybiel A. M. (1974). Visuo-cerebellar and cerebellar-visuo connections involving the ventral lateral geniculate nucleus. *Exp. Brain Res.* 20 303–306. 10.1007/BF00238320 4426354

[B46] Grodd W. Hülsmann E. Lotze M. Wildgruber D. Erb M. (2001). Sensorimotor mapping of the human cerebellum: fMRI evidence of somatotopic organization. *Hum. Brain Mapp.* 13 55–73. 10.1002/hbm.1025 11346886 PMC6871814

[B47] Herculano-Houzel S. (2010). Coordinated scaling of cortical and cerebellar numbers of neurons. *Front. Neuroanat.* 4:12 10.3389/fnana.2010.00012 PMC2839851 20300467

[B48] Herholz S. C. Halpern A. R. Zatorre R. J. (2012). Neuronal correlates of perception, imagery, and memory for familiar tunes. *J. Cogn. Neurosci.* 24 1382–1397. 10.1162/jocn_a_00216 22360595

[B49] Holcomb H. H. Medoff D. R. Caudill P. J. Zhao Z. Lahti A. C. Dannals R. F. (1998). Cerebral blood flow relationships associated with a difficult tone recognition task in trained normal volunteers. *Cereb. Cortex* 8 534–542. 10.1093/cercor/8.6.534 9758216

[B50] Houk J. C. Buckingham J. T. Barto A. G. (1996). Models of the cerebellum and motor learning. *Behav. Brain Sci.* 19 368–383. 10.1017/S0140525X00081474

[B51] Houk J. C. Keifer J. Barto A. G. (1993). Distributed motor commands in the limb premotor network. *Trends Neurosci.* 16 27–33. 10.1016/0166-2236(93)90049-R 7679234

[B52] Hsieh I.-H. Saberi K. (2007). Temporal integration in absolute pitch identification of absolute pitch. *Hear. Res.* 233 108–116. 10.1016/j.heares.2007.08.005 17919863

[B53] Huang C. M. Liu G. Huang R. (1982). Projections from the cochlear nucleus to the cerebellum. *Brain Res.* 244 1–8. 10.1016/0006-8993(82)90897-6 7116161

[B54] Hutchinson S. Lee L. H. Gaab N. Schlaug G. (2003). Cerebellar volume of musicians. *Cereb. Cortex* 13 943–949. 10.1093/cercor/13.9.943 12902393

[B55] Imamizu H. Kuroda T. Miyauchi S. Yoshioka T. Kawato M. (2003). Modular organization of internal models of tools in the human cerebellum. *Proc. Natl. Acad. Sci. U.S.A.* 100 5461–5466. 10.1073/pnas.0835746100 12704240 PMC154367

[B56] Ito M. (1970). Neurophysiological aspects of the cerebellar motor control system. *Int. J. Neurol.* 7 162–176. 5499516

[B57] Ito M. (2006). Cerebellar circuitry as a neuronal machine. *Prog. Neurobiol.* 78 272–303. 10.1016/j.pneurobio.2006.02.006 16759785

[B58] Ito M. (2008). Control of mental activities by internal models in the cerebellum. *Nat. Rev. Neurosci.* 9 304–313. 10.1038/nrn2332 18319727

[B59] Ito M. Sakurai M. Tongroach P. (1982). Climbing fibre induced depression of both mossy fibre responsiveness and glutamate sensitivity of cerebellar Purkinje cells. *J. Physiol.* 324 113–134. 10.1113/jphysiol.1982.sp014103 7097592 PMC1250696

[B60] Ivry R. B. Keele S. W. Diener H. C. (1988). Dissociation of the lateral and medial cerebellum in movement timing and movement execution. *Exp. Brain Res.* 73 167–180. 10.1007/BF00279670 3208855

[B61] Kawato M. Furukawa K. Suzuki R. (1987). A hierarchical neural network model for the control and learning of voluntary movements. *Biol. Cybern.* 56 1–17. 10.1007/bf00364149 3676355

[B62] Kellermann T. Regenbogen C. De Vos M. Mößnang C. Finkelmeyer A. Habel U. (2012). Effective connectivity of the human cerebellum during visual attention. *J. Neurosci.* 32 11453–11460. 10.1523/JNEUROSCI.0678-12.2012 22895727 PMC6621198

[B63] Kelly R. M. Strick P. L. (2003). Cerebellar loops with motor cortex and prefrontal cortex of a nonhuman primate. *J. Neurosci.* 23 8432–8444. 12968006 10.1523/JNEUROSCI.23-23-08432.2003 PMC6740694

[B64] Kleber B. Birbaumer N. Veit R. Trevorrow T. Lotze M. (2007). Overt and imagined singing of an Italian aria. *Neuroimage* 36 889–900. 10.1016/j.neuroimage.2007.02.053 17478107

[B65] Konnerth A. Llano I. Armstrong C. M. (1990). Synaptic currents in cerebellar Purkinje cells. *Proc. Natl. Acad. Sci. U.S.A.* 87 2662–2665. 10.1073/pnas.87.7.2662 1969639 PMC53750

[B66] Konoike N. Kotozaki Y. Miyachi S. Miyauchi C. M. Yomogida Y. Akimoto Y. (2012). Rhythm information represented in the fronto-parieto-cerebellar motor system. *Neuroimage* 63 328–338. 10.1016/j.neuroimage.2012.07.002 22796994

[B67] Kotz S. A. Schwartze M. (2010). Cortical speech processing unplugged: a timely subcortico-cortical framework. *Trends Cogn. Sci.* 14 392–399. 10.1016/j.tics.2010.06.005 20655802

[B68] Kraus N. Chandrasekaran B. (2010). Music training for the development of auditory skills. *Nat. Rev. Neurosci.* 11 599–605. 10.1038/nrn2882 20648064

[B69] Langner G. Schreiner C. E. (1988). Periodicity coding in the inferior colliculus of the cat: I. Neuronal mechanisms. *J. Neurophysiol.* 60 1799–1822. 3236052 10.1152/jn.1988.60.6.1799

[B70] Leiner H. C. Leiner A. L. Dow R. S. (1989). Reappraising the cerebellum: what does the hindbrain contribute to the forebrain? *Behav. Neurosci.* 102 998–1008. 10.1037/0735-7044.103.5.998 2679667

[B71] Lesage E. Morgan B. E. Olson A. C. Meyer A. S. Miall R. C. (2012). Cerebellar rTMS disrupts predictive language processing. *Curr. Biol.* 22 R794–R795. 10.1016/j.cub.2012.07.006 23017990 PMC3459089

[B72] Lindblom B. (1996). Role of articulation in speech perception: clues from production. *J. Acoust. Soc. Am.* 99 1683–1692. 10.1121/1.414691 8819859

[B73] Lingenhöhl K. Friauf E. (1994). Giant neurons in the rat reticular formation: a sensorimotor interface in the elementary acoustic startle circuit? *J. Neurosci.* 14 1176–1194. 8120618 10.1523/JNEUROSCI.14-03-01176.1994 PMC6577542

[B74] Liu T. Xu D. Ashe J. Bushara K. (2008). Specificity of inferior olive response to stimulus timing. *J. Neurophysiol.* 100 1557–1561. 10.1152/jn.00961.2007 18632890 PMC2544464

[B75] Llinas R. Baker R. Sotelo C. (1974). Electrotonic coupling between neurons in cat inferior olive. *J. Neurophysiol.* 37 560–571. 4827022 10.1152/jn.1974.37.3.560

[B76] Logan C. A. Fulk K. R. (1984). Differential responding to spring and fall song in mockingbird (*Mimus polyglottos*). *J. Comp. Psychol.* 98 3–9. 10.1037/0735-7036.98.1.3

[B77] Maess B. Koelsch S. Gunter T. C. Friederici A. D. (2001). Musical syntax is processed in Broca’s area: an MEG study. *Nat. Neurosci.* 4 540–545. 11319564 10.1038/87502

[B78] Mahon B. Z. Caramazza A. (2008). A critical look at the embodied cognition hypothesis and a new proposal for grounding conceptual content. *J. Physiol. Paris* 102 59–70. 10.1016/j.jphysparis.2008.03.004 18448316

[B79] Manley G. A. (2002). Evolution of structure and function of the hearing organ of lizards. *J. Neurobiol.* 53 202–211. 10.1002/neu.10115 12382276

[B80] Manni E. Petrosini L. (2004). A century of cerebellar somatotopy: a debated representation. *Nat. Rev. Neurosci.* 5 241–249. 10.1038/nrn1347 14976523

[B81] Marien P. Engelborghs S. Fabbro F. De Deyn P. P. (2001). The lateralized linguistic cerebellum: a review and a new hypothesis. *Brain Lang.* 79 580–600. 10.1006/brln.2001.2569 11781058

[B82] Marr D. (1969). A theory of cerebellar cortex. *J. Physiol.* 202 437–470. 10.1113/jphysiol.1969.sp008820 5784296 PMC1351491

[B83] Mathiak K. Hertrich I. Grodd W. Ackermann H. (2004). Discrimination of temporal information at the cerebellum: functional magnetic resonance imaging of nonverbal auditory memory. *Neuroimage* 21 154–162. 10.1016/j.neuroimage.2003.09.036 14741652

[B84] McLachlan N. Marco D. Light M. Wilson S. (2013). Consonance and pitch. *J. Exp. Psychol.* 142 1142–1158. 10.1037/a0030830 23294344

[B85] McLachlan N. M. (2009). A computational model of human pitch strength and height judgments. *Hear. Res.* 249 23–35. 10.1016/j.heares.2009.01.003 19271312

[B86] McLachlan N. M. (2011). A neurocognitive model of recognition and pitch segregation. *J. Acoust. Soc. Am.* 130 2845–2854. 10.1121/1.3643082 22087913

[B87] McLachlan N. M. Grayden D. B. (2014). Enhancement of speech perception in noise by periodicity processing: a neurobiological model and signal processing algorithm. *Speech Commun.* 57C, 114–125. 10.1016/j.specom.2013.09.007

[B88] McLachlan N. M. Marco D. J. T. Wilson S. J. (2013). The musical environment and auditory plasticity: hearing the pitch of percussion. *Front. Psychol.* 4:768 10.3389/fpsyg.2013.00768 PMC3807563 24187543

[B89] McLachlan N. M. Wilson S. W. (2010). The central role of recognition in auditory perception: a neurobiological model. *Psychol. Rev.* 117 175–196. 10.1037/a0018063 20063967

[B90] Meddis R. O’Mard L. P. (2006). Virtual pitch in a computational physiological model. *J. Acoust. Soc. Am.* 120 3861–3869. 10.1121/1.2372595 17225413

[B91] Medina J. F. Garcia K. S. Nores W. L. Taylor N. M. Mauk M. D. (2000). Timing mechanisms in the cerebellum: testing predictions of a large-scale computer simulation. *J. Neurosci.* 20 5516–5525. 10884335 10.1523/JNEUROSCI.20-14-05516.2000 PMC6772322

[B92] Medina J. F. Mauk M. D. (2000). Computer simulation of cerebellar information processing. *Nat. Neurosci.* 8 1205–1211. 10.1038/81486 11127839

[B93] Moberget T. Gullesen E. H. Andersson S. Ivry R. B. Endestad T. (2014). Generalized role for the cerebellum in encoding internal models: evidence from semantic processing. *J. Neurosci.* 34 2871–2878. 10.1523/JNEUROSCI.2264-13.2014 24553928 PMC3931501

[B94] Moberget T. Ivry R. B. (2016). Cerebellar contributions to motor control and language comprehension: searching for common computational principles. *Ann. N. Y. Acad. Sci.* 1369 154–171. 10.1111/nyas.13094 27206249 PMC5260470

[B95] Moore J. W. Desmond J. E. Berthier N. E. (1989). Adaptively timed conditioned responses and the cerebellum: a neural network approach. *Biol. Cybern.* 62 17–28. 10.1007/BF00217657 2590676

[B96] Murdoch B. E. (2010). The cerebellum and language: historical perspective and review. *Cortex* 46 858–868. 10.1016/j.cortex.2009.07.018 19828143

[B97] O’Halloran C. J. Kinsella G. J. Storey E. (2012). The cerebellum and neuropshycological functioning: a critical review. *J. Clin. Exp. Neuropsychol.* 34 35–56. 10.1080/13803395.2011.614599 22047489

[B98] Ohyama T. Nores W. L. Murphy M. Mauk M. D. (2003). What the cerebellum computes. *Trends Neurosci.* 26 222–227. 10.1016/S0166-2236(03)00054-7 12689774

[B99] Oka H. Jinnai K. Yamamoto T. (1979). The parieto-rubro-olivary pathway in the cat. *Exp. Brain Res.* 37 115–125. 10.1007/BF01474258 488210

[B100] Paré D. Steriade M. Deschênes M. Bouhassira D. (1990). Prolonged enhancement of anterior thalamic synaptic responsiveness by stimulation of a brain-stem cholinergic group. *J. Neurosci.* 10 20–33. 2299393 10.1523/JNEUROSCI.10-01-00020.1990 PMC6570342

[B101] Parsons L. M. Petacchi A. Schmahmann J. D. Bower J. M. (2009). Pitch discrimination in cerebellar patients: evidence for a sensory deficit. *Brain Res.* 1303 84–96. 10.1016/j.brainres.2009.09.052 19766609

[B102] Perrett S. P. Ruiz B. P. Mauk M. D. (1993). Cerebellar cortex lesions disrupt learning-dependent timing of conditioned eyelid responses. *J. Neurosci.* 13 1708–1718. 8463846 10.1523/JNEUROSCI.13-04-01708.1993 PMC6576722

[B103] Petacchi A. Laird A. R. Fox P. T. Bower J. M. (2005). Cerebellum and auditory function: an ALE meta-analysis of functional neuroimaging studies. *Hum. Brain Mapp.* 25 118–128. 10.1002/hbm.20137 15846816 PMC6871682

[B104] Phelps E. A. (2004). Human emotion and memory: interactions of the amygdala and hippocampus complex. *Curr. Opin. Neurobiol.* 14 198–202. 10.1016/j.conb.2004.03.015 15082325

[B105] Poldrack R. A. Clark J. Paré-Blagoev E. J. Shohamy D. Creso Moyano J. Myers C. (2001). Interactive memory systems in the human brain. *Nature* 414 546–550. 10.1038/35107080 11734855

[B106] Ramnani N. (2006). The primate cortico-cerebellar system: anatomy and function. *Nat. Rev. Neurosci.* 7 511–522. 10.1038/nrn1953 16791141

[B107] Ramnani N. Behrens T. E. Johansen-Berg H. Richter M. C. Pinsk M. A. Andersson J. L. (2006). The evolution of prefrontal inputs to the cortico-pontine system: diffusion imaging evidence from Macaque monkeys and humans. *Cereb. Cortex* 16 811–818. 10.1093/cercor/bhj024 16120793

[B108] Rand R. W. (1954). An anatomical and experimental study of the cerebellar nuclei and their efferent pathways in the monkey. *J. Comp. Neurol.* 101 167–223. 10.1002/cne.901010107 13211856

[B109] Rauschecker J. P. Tian B. (2000). Mechanisms and streams for processing of “what” and “where” in auditory cortex. *Proc. Natl. Acad. Sci. U.S.A.* 97 11800–11806. 10.1073/pnas.97.22.11800 11050212 PMC34352

[B110] Ravizza S. M. McCormick C. A. Schlerf J. E. Justus T. Ivry R. B. Fiez J. A. (2006). Cerebellar damage produces selective deficits in verbal working memory. *Brain* 129 306–320. 10.1093/brain/awh685 16317024

[B111] Reese N. B. Garcia-Rill E. Skinner R. D. (1995). Auditory input to the pedunculopontine nucleus: I. evoked potentials. *Brain Res. Bull.* 37 257–264. 10.1016/0361-9230(95)00002-V 7627568

[B112] Roberts P. D. Portfors C. V. (2008). Design principles of sensory processing in cerebellum-like structures: early stage processing of electrosensory and auditory objects. *Biol. Cybern.* 98 491–507. 10.1007/s00422-008-0217-1 18491162

[B113] Roll M. Mårtensson F. Sikström S. Apt P. Arnling-Bååth R. Horne M. (2012). Atypical associations to abstract words in Broca’s aphasia. *Cortex* 48 1068–1072. 10.1016/j.cortex.2011.11.009 22172978

[B114] Sacchetti B. Scelfo B. Strata P. (2005). The cerebellum: synaptic changes and fear conditioning. *Neuroscientist* 11 217–227. 10.1177/1073858405276428 15911871

[B115] Salimpoor V. N. van den Bosch I. Kovacevic N. McIntosh A. R. Dagher A. Zatorre R. J. (2012). Interactions between the nucleus accumbens and auditory cortices predict music reward value. *Science* 340 216–219. 10.1126/science.1231059 23580531

[B116] Salmond C. H. Vargha-Khadem F. Gadian D. G. de Haan M. Baldeweg T. (2007). Heterogeneity in the patterns of neural abnormality in autistic spectrum disorders: evidence from ERP and MRI. *Cortex* 43 686–699. 10.1016/S0010-9452(08)70498-2 17710821

[B117] Schlösser R. Hutchinson M. Joseffer S. Rusinek H. Saarimaki A. Stevenson J. (1998). Functional magnetic resonance imaging of human brain activity in a verbal fluency task. *J. Neurol. Neurosurg. Psychiatry* 64 492–498. 10.1136/jnnp.64.4.492 9576541 PMC2170033

[B118] Schmahmann J. D. Sherman J. C. (1998). The cerebellar cognitive affective syndrome. *Brain* 121 561–579. 10.1093/brain/121.4.561 9577385

[B119] Schwartz C. Their P. (1999). Binding of signals relevant for action: towards a hypothesis of the functional role of the pontine nuclei. *Trends Neurosci.* 22 443–451. 10.1016/S0166-2236(99)01446-0 10481191

[B120] Schwartze M. Kotz S. A. (2016). Contributions of cerebellar event-based temporal processing and preparatory function to speech perception. *Brain Lang.* 161 28–32. 10.1016/j.bandl.2015.08.005 26362972

[B121] Seither-Preisler A. Johnson L. Krumbholz K. Nobbe A. Patterson R. Seither S. (2007). Tone sequences with conflicting fundamental pitch and timbre changes are heard differently by musicians and nonmusicians. *J. Exp. Psychol.* 33 743–751. 10.1037/0096-1523.33.3.743 PMC2821799 17563235

[B122] Sens P. M. de Almeida C. I. (2007). Participation of the cerebellum in auditory processing. *Rev. Bras. Otorrinolaringol.* 73 266–270. 10.1590/S0034-72992007000200019 PMC9452195 17589737

[B123] Snider R. S. Eldred E. (1951). Cerebro-cerebellar relationships in the monkey. *J. Neurophysiol.* 15 27–40. 10.1152/jn.1952.15.1.27 14908622

[B124] Snider R. S. Stowell A. (1944). Receiving areas of tactile, auditory and visual systems in the cerebellum. *J. Neurophysiol.* 7 331–357.

[B125] Sokolov A. A. Erb M. Grodd W. Pavlova M. A. (2014). Structural loop between the cerebellum and the superior temporal sulcus: evidence from diffusion tensor imaging. *Cereb. Cortex* 24 626–632. 10.1093/cercor/bhs346 23169930

[B126] Stoodley C. J. Valera E. M. Schmahmann J. D. (2012). Functional topography of the cerebellum for motor and cognitive tasks: an fMRI study. *Neuroimage* 59 1560–1570. 10.1016/j.neuroimage.2011.08.065 21907811 PMC3230671

[B127] Strick P. J. Dum R. P. Fiez J. A. (2009). Cerebellum and non-motor function. *Annu. Rev. Neurosci.* 32 413–434. 10.1146/annurev.neuro.31.060407.125606 19555291

[B128] Terhardt E. Stoll G. Seewann M. (1982). An algorithm for extraction of pitch and pitch salience from complex tonal signals. *J. Acoust. Soc. Am.* 71 679–688. 10.1121/1.387544 6699300

[B129] Thach W. T. (2007). On the mechanism of cerebellar contributions to cognition. *Cerebellum* 6 163–167. 10.1080/14734220701373530 17786811

[B130] Thompson R. F. (1986). The neurobiology of learning and memory. *Science* 233 941–947. 10.1126/science.3738519 3738519

[B131] Thompson R. F. Steinmetz J. E. (2009). The role of the cerebellum in classical conditioning of discrete behavioral responses. *Neuroscience* 162 732–755. 10.1016/j.neuroscience.2009.01.041 19409234

[B132] Tricomi E. Delgado M. R. McCandliss B. D. McClelland J. L. Fiez J. A. (2006). Performance feedback drives caudate activation in a phonological learning task. *J. Cogn. Neurosci.* 18 1029–1043. 10.1162/jocn.2006.18.6.1029 16839308

[B133] Tzounopoulos T. Kim Y. Oertel D. Trussell L. O. (2004). Cell-specific, spike timing-dependent plasticities in the dorsal cochlear nucleus. *Nat. Neurosci.* 7 719–725. 10.1038/nn1272 15208632

[B134] Van Der Giessen R. S. Koekkoek S. K. van Dorp S. De Gruijl J. R. Cupido A. Khosrovani S. (2008). Role of olivary electrical coupling in cerebellar motor learning. *Neuron* 58 599–612. 10.1016/j.neuron.2008.03.016 18498740

[B135] Vandervert L. (2011). The evolution of language: the cerebro-cerebellar blending of visual-spatial working memory with vocalizations. *J. Mind Behav.* 32 317–332.

[B136] Vitousek M. N. Adelman J. S. Gregory N. C. Clair J. J. (2007). Heterospecific alarm call recognition in a non-vocal reptile. *Biol. Lett.* 3 632–634. 10.1098/rsbl.2007.0443 17911047 PMC2391237

[B137] Weaver A. (2005). Reciprocal evolution of the cerebellum and neocortex in fossil humans. *Proc. Natl. Acad. Sci. U.S.A.* 102 3576–3580. 10.1073/pnas.0500692102 15731345 PMC553338

[B138] Weinberger N. M. (2011). The medial geniculate, not the amygdala, as the root of auditory fear conditioning. *Hear. Res.* 274 61–74. 10.1016/j.heares.2010.03.093 20466051 PMC2949681

[B139] Welsh J. P. Lang E. J. Suglhara I. Llinás R. (1995). Dynamic organization of motor control within the olivocerebellar system. *Nature* 374 453–457. 10.1038/374453a0 7700354

[B140] Wieser H. Mazzola G. (1986). Musical consonances and dissonances: are they distinguished independently by the right and left hippocampi? *Neuropsychologia* 24 805–812. 3808288 10.1016/0028-3932(86)90079-5

[B141] Wilson S. J. Lusher D. Martin C. L. Rayner G. McLachlan N. (2012). Intersecting factors lead to absolute pitch acquisition that is maintained in a “fixed do” environment. *Music Percept.* 29 285–296. 10.1525/mp.2012.29.3.285

[B142] Wilson S. J. Lusher D. Wan C. Y. Dudgeon P. Reutens D. C. (2009). The neurocognitive components of pitch processing: insights from absolute pitch. *Cereb. Cortex* 19 724–732. 10.1093/cercor/bhn121 18663250 PMC2638817

[B143] Wolpert D. M. Miall R. C. Kawato M. (1998). Internal models in the cerebellum. *Trends Cogn. Sci.* 2 338–347. 10.1016/S1364-6613(98)01221-2 21227230

[B144] Wyneken J. (2007). Reptilian neurology: anatomy and function. *Vet. Clin. North Am.* 10 837–853. 10.1016/j.cvex.2007.05.004 17765850

[B145] Xiang H. Lin C. Ma X. Zhang Z. Bower J. M. Weng X. (2003). Involvement of the cerebellum in semantic discrimination: an fMRI study. *Hum. Brain Mapp.* 18 208–214. 10.1002/hbm.10095 12599279 PMC6872119

[B146] Yeo C. H. Hesslow G. (1998). Cerebellum and conditioned reflexes. *Trends Cogn. Sci.* 2 322–330. 10.1016/S1364-6613(98)01219-4 21227228

[B147] Zatorre R. J. Halpern A. R. (2005). Mental concerts: musical imagery and auditory cortex. *Neuron* 47 9–12. 10.1016/j.neuron.2005.06.013 15996544

[B148] Zettin M. Cappa S. F. D’Amico A. Rago R. Perino C. Perani D. (1997). Agrammatic speech production after a right cerebellar haemorrhage. *Neurocase* 3 375–380. 10.1080/13554799708411976

[B149] Zion Golumbic E. M. Ding N. Bickel S. Lakatos P. Schevon C. A. McKhann G. M. (2013). Mechanisms underlying selective neuronal tracking of attended speech at a “cocktail party.” *Neuron* 77 980–991. 10.1016/j.neuron.2012.12.037 23473326 PMC3891478

